# Hyperammonemic Encephalopathy in Multiple Myeloma: A Case Report

**DOI:** 10.7759/cureus.33626

**Published:** 2023-01-10

**Authors:** Ilma Vahora, Ezhil Panneerselvam, Abhizith Deoker

**Affiliations:** 1 Internal Medicine, Texas Tech University Health Sciences Center El Paso, El Paso, USA; 2 Internal Medicine, Texas Tech University Health Sciences Center El Paso Paul L. Foster School of Medicine, El Paso, USA

**Keywords:** cyclophosphamide, altered mental status, hyperammonemic encephalopathy, ammonia, multiple myeloma

## Abstract

Multiple myeloma (MM) typically presents as lytic bony lesions, hypercalcemia, anemia, and renal failure. Only a few cases of hyperammonemic encephalopathy (HE) attributed to multiple myeloma have been reported. We report a case of a 68-year-old Hispanic female diagnosed with multiple myeloma and presented with altered mental status and elevated ammonia levels found to have HE. The pathology behind HE is associated with higher ammonia levels produced by myeloma cell lines in the absence of liver disease. Due to the wide range of differentials for altered mental status (AMS), HE often gets missed and causes delayed treatment and the associated higher mortality. The primary treatment is chemotherapy. Lactulose and rifaximin must be initiated; however, it is ineffective if solely used. In our case, chemotherapy was not considered a treatment option in light of the patient's pancytopenia and infection. Our case is unique, as despite adequately treating other commonly suspected causes of AMS such as infection, there was no expected improvement in the patient's clinical status noticed, eventually leading to intubation due to worsening AMS. Given the patient's history of multiple myeloma, non-compliance with chemotherapy before presentation, and elevated ammonia levels raised suspicion for HE. Clinicians are encouraged to acquaint themselves with HE as a differential for patients presenting with MM flare and AMS, specifically when other potential causes of AMS are ruled out and addressed.

## Introduction

Multiple myeloma (MM) is a malignancy of immature plasma cell expansion and the production of abnormal immunoglobulin (Ig) causing clinical abnormalities such as anemia, renal insufficiency, hypercalcemia, or bone lesions [1]. Immunoglobulins are made of heavy chains like IgG, IgA, IgD, and IgM complexed with a light chain kappa (κ) or lambda (λ) [2]. The risk of infection increases due to the immature proliferation of immunoglobulin [1]. Monoclonal gammopathies are clonal expansion of plasma cells or lymphoplasmacytic cells, which forms a monoclonal spike [2]. Bone marrow plasmacytosis causes an osteolytic lesion in 60% of cases seen on imaging [1].

Hyperammonemic encephalopathy (HE) is a rare manifestation of multiple myeloma (MM), especially in the absence of more common causes of elevated ammonium levels such as liver disease [1]. Ammonium is an intermediate product of protein catabolism, which is eliminated as urea [1]. However, when excessive ammonium levels accumulate, it causes neurotoxicity, leading to altered mental status (AMS) [1]. In HE, one theory is that different MM cell lines produce increased amounts of ammonium [1,2]. Patients with HE usually respond well to treatment with chemotherapy such as cyclophosphamide, if initiated early [3]. We present the case of a Hispanic female who had rapidly progressing MM with the rare complication of HE. Authors exhibit this case to appraise HE as one of the differentials in patients with MM who present with altered mental status (AMS) and are suspected to have no other causes of elevated ammonia.

## Case presentation

A 68-year-old Hispanic female with a medical history of multiple myeloma presented with AMS for three to four days. At the age of 67, she was diagnosed with IgA kappa light chain subtype multiple myeloma when she presented with fractures of the T4 vertebral body, lytic bone lesions of the skull and axial spine, hypercalcemia, and anemia after she had a ground-level fall. Bone marrow biopsy at that time showed 80% abnormal plasma cells. Cytogenetics abnormalities by fluorescence in situ hybridization (FISH) showed deletion of t(4;14) but no deletion in 17P and a beta-2 microglobulin of 5.4 mg/L. Based on hematology-oncology recommendations, the patient was started on bortezomib, lenalidomide, and dexamethasone, along with zoledronic acid for hypercalcemia and supportive blood transfusions as appropriate. Apart from these, no other medications were initiated. However, the patient appeared to be non-compliant with the recommended regimen and likely stopped treatment at least 10 months prior to the second admission. About 14 months after the initial diagnosis, she presented to the hospital with acute onset of altered mental status. Vitals on admission showed temperature 36.6, blood pressure 126/76, respiratory rate (RR) 18, heart rate 80, and saturating 98% on room air. Pertinent physical findings included no icterus, jugular venous distension or ascites, awake, alert, and oriented x 0, Glasgow Coma Scale 13, and strength 5/5 in bilateral upper and lower extremities. Complete blood count showed pancytopenia with a white cell count of 2.31 mcL, hemoglobin of 7.0 g/dL, and platelets of 48 mcL. Metabolic profile was remarkable for corrected calcium of 14 mg/dl, bicarbonate of 17 mmol/L, blood urea nitrogen of 30 mg/dL, creatinine of 1.20 mg/dL, normal liver enzymes, and ammonia level elevated at 91.5 umol/l. Lastly, a urinalysis was concerning for urinary tract infection (UTI) - culture grew Enterococcus faecalis, but blood culture and chest X-ray depicted no infection source. On imaging, multiple lytic lesions and fractures to the calvarium, ribs, spine, and pelvis were noted. Computed tomography (CT) and MRI of the head were unremarkable except for osteolytic lesions. CT abdomen revealed normal liver morphology. Despite optimized supportive care with pamidronate, calcitonin, and fluid hydration leading to improvement in hypercalcemia, lactulose, and rifaximin for ammonia and piperacillin-tazobactam antibiotics for UTI, the patient continued to exhibit worsening mentation and repeat ammonia was found to be 107. Due to decreased mentation and concern about airway protection, the patient was required to be intubated on Day 5 of admission and passed away soon after.

## Discussion

Multiple myeloma is described as a neoplastic proliferation of immunoglobulin-producing plasma cells comprising 10% of all hematologic malignancies [3]. It is more common in men and individuals of African American descent [3]. The diagnosis of MM is created based on the presence of either one or more myeloma-defining events (MDE) in addition to evidence of either 10% or more clonal plasma cells on bone marrow examination or a biopsy-proven bony or soft tissue plasmacytoma [4]. MDE consists of established CRAB (hypercalcemia, renal failure, anemia, or lytic bone lesions) features and three biomarkers associated with near inevitable progression to end-organ damage: clonal bone marrow plasma cells ≥60%, serum free light chain (FLC) ratio ≥100 (provided involved FLC level is ≥100 mg/L), and more than one focal lesion on MRI [4]. The monoclonal protein can be detected by serum-free light chain analysis or protein electrophoresis of the serum (SPEP) or urine (UPEP) [3,4]. The imaging usually shows classic multiple, small, well-circumscribed, lytic, punched-out round lesions within the skull, spine, and pelvis [4].

MM can present with a wide range of symptoms weight loss, bone anemia, generalized weakness, and neurologic manifestations, namely extradural spinal cord compression, radiculopathy, and peripheral neuropathy [5]. One of the rare presentations of multiple myeloma is hyperammonemic encephalopathy in the absence of liver impairment [6]. Only a few cases of HE secondary to MM have been reported so far [6]. HE, in association with MM, is a life-threatening condition with a mortality rate of 44% [6,7]. It is due to an accumulation of ammonium, which manifests as altered mental status, respiratory alkalosis, coma, and death [7]. The patient with MM did not have hepatic dysfunction, hence it was not the source of hyperammonemia [2,5]. Other noted causes of hyperammonemia are medications such as valproate, 5 fluorouracil, and Apatinib [8,9]. Certain urase-producing organisms, such as proteus mirabilis, Escherichia (E.) coli, Klebsiella pneumonia, Pseudomonas, and Helicobacter (H.) pylori were also known to cause elevated ammonia levels and HE, which had not been seen in our patient [10]. The mechanism behind chemotherapy-induced HE is poorly understood [9]. However, valproic acid inhibits the activity of carbamoyl phosphate synthetase and hence decreases the excretion of ammonia and raises plasma ammonia levels [8].

The exact mechanism of HE is unclear; however, based on the studies published so far, it is proposed that ammonia is produced and released from myeloma cell lines or myeloma-related humoral factors influence amino acid metabolism, leading to hyperammonemia and its complications as seen in our patient [2,11]. The mainstay of treatment for HEMM is chemotherapy, which includes dexamethasone, high-dose cyclophosphamide, and bortezomib [11,12]. Lactulose and hemodialysis do not lower ammonium levels in a sustained manner without concurrent chemotherapy [2,11]. Unfortunately, chemotherapy could not be started on this patient due to her rapid decline, infection, and pancytopenia. Pham et al. mentioned in their study that in-patient mortality for HE was 31% in patients who received MM-directed therapy and 100% in those who did not receive MM-directed treatment [12]. Delays or contraindications of chemotherapy make prognosis poor and associated unconscionable mortality risk [2,12].

## Conclusions

HE is an extremely rare manifestation of multiple myeloma and is associated with poor outcomes if not treated properly. Our patient had UTI and hypercalcemia, which are common causes of AMS in the elderly. Despite properly addressed treatment, the worsening of the patients' AMS was noticed, leading to the least common cause of AMS, HE, as the reason for AMS. This article should bring the clinician's attention to considering such unlikely causes as differentials and prompt treatment accordingly, even if the most common causes of AMS are present and no improvement is seen after appropriate treatment.
